# Use Case Evaluation and Digital Workflow of Breast Cancer Care by Artificial Intelligence and Blockchain Technology Application

**DOI:** 10.3390/healthcare10102100

**Published:** 2022-10-20

**Authors:** Sebastian Griewing, Michael Lingenfelder, Uwe Wagner, Niklas Gremke

**Affiliations:** 1 Institute for Healthcare Management, Chair of General Business Administration, Philipps-University Marburg, Universitätsstraße 24, 35037 Marburg, Germany; 2 Department of Gynecology and Obstetrics, University Hospital Marburg, Philipps-University Marburg, Baldingerstraße, 35043 Marburg, Germany; 3 Commission for Digital Medicine, German Association of Gynecology and Obstetrics DGGG, Jägerstraße 58-60, 10117 Berlin, Germany

**Keywords:** artificial intelligence, distributed ledger technology, blockchain, ovarian cancer, breast cancer, university hospital

## Abstract

This study aims at evaluating the use case potential of breast cancer care for artificial intelligence and blockchain technology application based on the patient data analysis at Marburg University Hospital and, thereupon, developing a digital workflow for breast cancer care. It is based on a retrospective descriptive data analysis of all in-patient breast and ovarian cancer patients admitted at the Department of Gynecology of Marburg University Hospital within the five-year observation period of 2017 to 2021. According to the German breast cancer guideline, the care workflow was visualized and, thereon, the digital concept was developed, premised on the literature foundation provided by a Boolean combination open search. Breast cancer cases display a lower average patient case complexity, fewer secondary diagnoses, and performed procedures than ovarian cancer. Moreover, 96% of all breast cancer patients originate from a city with direct geographical proximity. Estimated circumference and total catchment area of ovarian present 28.6% and 40% larger, respectively, than for breast cancer. The data support invasive breast cancer as a preferred use case for digitization. The digital workflow based on combined application of artificial intelligence as well as blockchain or distributed ledger technology demonstrates potential in tackling senological care pain points and leveraging patient data safety and sovereignty.

## 1. Introduction

As technological innovation continues to transform the world, many areas of daily life have changed and become faster, more efficient, or simply easier. Technological progress shapes whole businesses and industries, but in healthcare, the mills often grind slowly, sticking to the status quo. Patient data are predominantly stored in data silos of physicians’ offices or hospitals, hindering interoperability and data exchange between healthcare stakeholders. The status quo holds on to outdated and insecure technologies as faxing machines and e-mail remain to be an integral method used for sharing sensitive patient data or medical health records [1,2]. Literature supports the use of artificial intelligence and blockchain, or more precisely, distributed ledger technology (DLT), in healthcare to counteract the aforementioned issues that render patient care slow, cumbersome, resource-intensive, and ultimately costly [3,4,5,6,7,8]. Within healthcare, blockchain technology has been identified as a viable solution for the future to enable secure and fast data sharing [3]. Beyond that, its properties have been acknowledged to be able to contribute to the United Nations’ Sustainable Development Goals (SDGs) [9]. However, the question of whether applicability can be confirmed for all medical sub-specialties remains open. In oncology, large amounts of data are produced on a daily basis owing to the chronic progression of cancer disease and the broad network of involved stakeholders within diagnosis, treatment, and follow-up care [2]. There is a special need for increased efficiency in data sharing to keep pace with the growing number of cancer cases and the associated high amounts of data [1,10]. During the previous year of 2021, the German Association of Gynecology and Obstetrics (DGGG) founded the “Commission for Digital Medicine”, a devotion to accelerating the digitization of gynecological and obstetric care. The working group takes aim at promoting the development of interdisciplinary collaboration between the scientific disciplines of medicine, economics, and information technology to tackle present and future challenges of gynecological and obstetric care. The focus of the efforts is set on driving patient centrism and empowerment while enhancing data safety and the efficiency of the medical service provision to lift obligations from the involved providers and set free working time for empathic and patient-oriented caregiving via integration of modern information technologies.

This study aims at evaluating the use case potential of breast cancer care and, thereon, developing a concept for the application of artificial intelligence and blockchain or distributed ledger technology for the digital workflow of breast cancer care. Therefore, it is based on an analysis of the regional breast cancer care network of Marburg University Hospital to link the concept with real care data. A comparison with the data of ovarian cancer care aims to elaborate the preferred use case potential of breast cancer within oncology and gyne-oncology. The study aims at the scientifically sound identification of the current and future challenges of senological oncological care as the most prominent sub-group of oncology and gyne-conology and the response on how the present patient journey can be modified by artificial intelligence and distributed ledger technology application. The study concludes on phrasing the scientific gap that needs to be filled by further research efforts to launch a successful pilot project in senological care.

## 2. Methods

### 2.1. Statistical Methods

The analysis is based on a retrospective data evaluation of all in-patiently treated invasive breast cancer (C50) and invasive ovarian cancer (C56) patients admitted at the Department of Gynecology and Obstetrics of Marburg University Hospital within the five-year observation period of 1 January 2017 to 31 December 2021. Data evaluation is methodologically based on descriptive statistical assessment. Therefore, the study data analysis focuses on the distribution of age and gender, yearly development of the total patient number, primary and secondary International Classification of Disease (ICD) diagnoses, corresponding two- and five-digit zip code frequency, and geographical distribution, as well as basic economic parameters of revenue and cost accounting, length of stay, and the patient clinical complexity level (PCCL).

### 2.2. Patient Selection

The case-related treatment and health economic performance data of n = 2592 cases of the Department of Gynecology and Obstetrics of Marburg University Hospital were recorded within the mentioned observation period. The total case count divides into the two sub-groups of invasive breast cancer (C50) and invasive ovarian cancer (C56) by n_C50_ = 2189 and n_C56_ = 403, accordingly. To avoid violation of the compliance and ethics guidelines of Marburg University Hospital, the data were anonymized and no patient-specific case numbers were used.

### 2.3. Data Analysis

The data for the analysis and comparison of the basic clinical and health economic performance parameters of the C50 and C56 cases were retrieved from the hospital performance controlling program QlikView^®^, which registers all services and procedures entered into the hospital information system during hospitalization. The observation period was selected for 1 January 2017 to 31 December 2021. The QlikView^®^ software, developed by the company QlikTech (Radnor, PA, USA), offers the possibility of displaying the clinical treatment services in connection with the corresponding health economic key figures both in the form of raw data and as interactive analyses. Figures were generated using QlikView^®^ and then processed based on descriptive statistics methods using Excel^®^ (Version 16.65, Microsoft Corporation, Redmond, WA, USA). For visualization and analysis of the geographical case distribution, the case-related zip codes of observation year 2021 were mapped using the MyMaps^®^ tool of Google LLC (Mountain View, CA, USA) according to frequency of occurrence. The circumference and total catchment area for each sub-group were estimated using the MyMaps^®^ measurement tools. In order to provide enhanced visualization clarity, zip codes with only one recorded patient were excluded for C50 invasive breast cancer (n_C50=1_). Furthermore, zip codes outliers with a direct geographical distance over 100 km to Marburg University Hospital were excluded from the C50 (n_C50>100km_) and C56 (n_C56>100km_) mapping process.

### 2.4. Concept Development

The literature for the concept development was identified by a Boolean combination open search performed for {(“Artificial Intelligence” OR “Machine Learning”) AND (“Medicine” OR Oncology)} and {(Distributed Ledger OR Blockchain) AND (Medicine OR Oncology)}. Inclusion was assessed and performed upon a time horizon of 2011 or later, English or German language, white paper format, and authorship by academic and political institutions or multinational companies in the fields of information technology, medical technology or auditing, risk, tax, and financial consulting or advisory service. The technological and digital workflow was developed accordingly.

The visualization of the breast cancer workflow is based on the version 4.4 S3 guideline “Evidence-based Guideline for the Early Detection, Diagnosis, Treatment and Follow-up of Breast Cancer” from May 2021 of the German Guideline Program Oncology (GGPO) of the German Cancer Association (DKG) and German Association for Gynecology and Obstetrics (DGGG). The description focuses on common primary invasive mamma carcinoma diagnosis and excludes the specific cases of recurrent, metastatic, and familial breast cancer.

## 3. Results

### 3.1. Patient Data Analysis

#### 3.1.1. Descriptive Analysis

Within the selected observation period, a total of n_C50_ = 2189 patients with primary diagnosis C50 invasive breast cancer and a total of n_C56_ = 403 of C56 ovarian cancer cases were admitted to the department of Gynecology and Obstetrics of the University Hospital Marburg. The related results of the analyzed patient data are illustrated in Table 1.

#### 3.1.2. Geographical and Frequency Distribution

Table 2 depicts the geographical zip code distribution based on the first two digits as well as the corresponding counties. The majority of 78% of all patients originate from a city with a zip code starting with the digits 35, belonging to the counties of Marburg-Biedenkopf, Waldeck-Frankenberg, Gießen, Vogelsbergkreis, Lahn-Dill-Kreis, Wetteraukreis, and Limburg-Weilburg. Five cases did not have any registered zip code.

**Figure 1:**
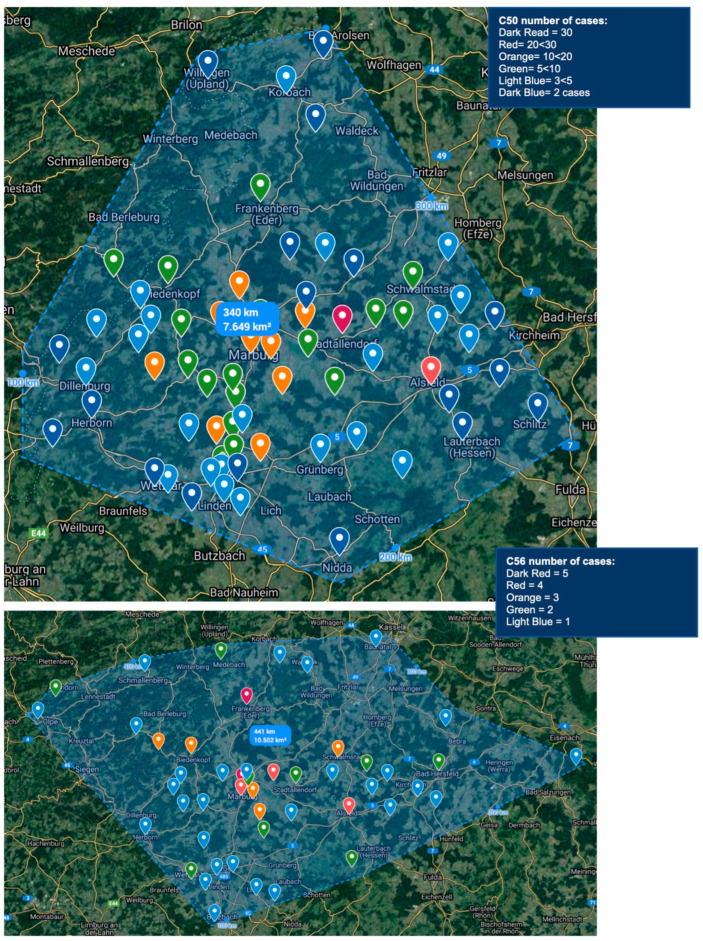
C50 and C56 geographical distribution by five-digit zip codes.

Furthermore, the regional five-digit zip code distribution of the C50 patients is depicted in Figure 1 in comparison with the C56 cases. For breast cancer mapping, a total of n_C50=1_ = 35 and n_C50>100 km_ = 1 cases were excluded from visualization, as well as n_C56>100km_ = 6 cases for ovarian cancer. The estimated circumference of the C50 cases amounts to 350 km and 450 km for C56, leaving an estimated total catchment of 7500 km^2^ and 10,500 km^2^ for breast and ovarian cancer, respectively.

### 3.2. Breast Cancer Care Workflow

**Figure 2:**
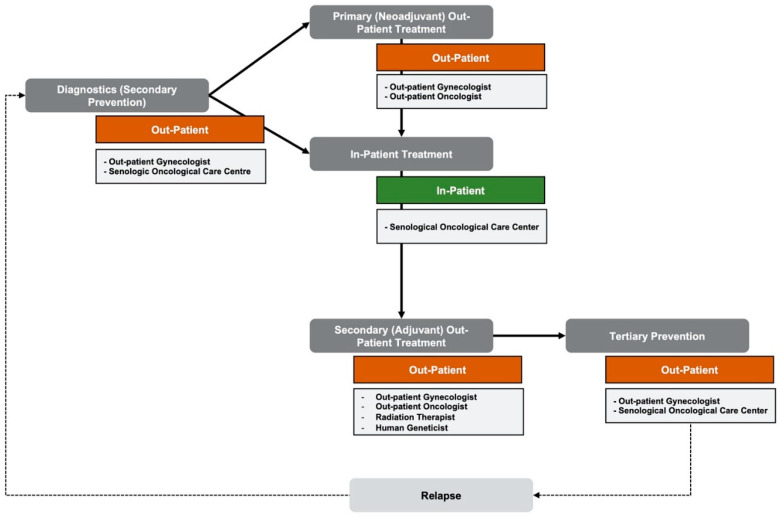
Overview of the breast cancer care workflow based on the German guideline.

The care workflow is depicted in a holistic manner based on the German S3 guideline “Evidence-based Guideline for the Early Detection, Diagnosis, Treatment, and Follow-Up of Breast Cancer”. The workflow is built up by a close-knit out-patient secondary prevention system, leading into primary out-patient neoadjuvant therapy or primary in-patient treatment based on the diagnostics’ outcome and medical history of the patient. Once primary neoadjuvant treatment is finished, it concludes in in-patient treatment at a certified senological oncological care center. In the case of malignant invasive breast carcinoma, a secondary out-patient adjuvant patient treatment will follow, leading into another close-knit tertiary prevention system facilitated in out-patient manner. This tertiary prevention will prevail for at least 10 years after primary diagnosis to enable early detection of relapse. Figure 2 depicts the overview of the breast cancer workflow.

### 3.3. Technological Concept

#### 3.3.1. Applied Literature

The literature used for concept development and the following discussion were identified by a Boolean combination open search performed for {(“Artificial Intelligence” OR “Machine Learning”) AND (Medicine OR Oncology)} and {(“Distributed Ledger” OR Blockchain) AND (Medicine OR Oncology)}. Upon application of the previously presented inclusion criteria, a total of n_DLT_ = 15 and n_AI_ = 19 industry reports and white papers were selected. The results of the Boolean combination search are stated in Table 3.

#### 3.3.2. Artificial Intelligence Component

Artificial intelligence can be described as a system’s ability to correctly interpret external data, learn from such data, and use those learnings to achieve tasks via flexible adaption [6,8,28,43,44]. In the case of direct patient care, the application of artificial intelligence principles can be grouped into machine learning and natural language processing. Thus, machine learning streamlines structured data such as imaging, medical history, or histopathologic results to attempt to cluster a patient’s traits and act upon it, while natural language processing methods extract information from unstructured data to supply addition to available data settings [28,45]. Therefore, the potential for artificial intelligence in healthcare is predominantly seen in leveraging performance and efficiency, enhancing clinical decision quality, actively managing specific health populations, and empowering patients and providers [6,8,28,43,44,46,47]. As such, an artificial intelligence system is able to evaluate masses of data through algorithmic analysis to encompass automation [48].

The hereby presented technological concept integrates artificial intelligence principles by creating a digital companion designed to fulfill the needs of the patient to enable efficient navigation through individual treatment and disease monitoring and provide support for the provider through partial automation of the workflow and digital support for decision making.

#### 3.3.3. Blockchain or Distributed Ledger Technology Component

**Figure 3:**
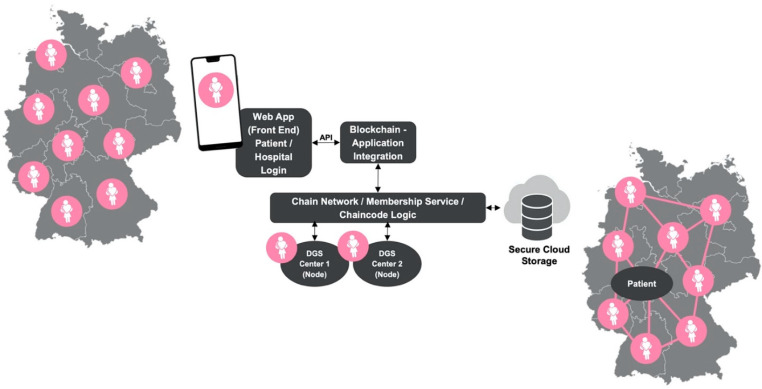
Overview of the technological solution (DGS = German senological center, API = application programming interface).

The technological data sharing concept is built upon a blockchain and distributed ledger technology solution. Fundamentally, a hybrid data management approach will be realized with metadata stored on-chain, while actual sensitive patient data are encrypted and stored on an off-chain, privacy-compliant cloud-storage [49,50]. Metadata itself can lead to the off-chain patient data, but itself is unreadable and gibberish. To comply with GDPR ruling and assure data privacy and security, the concept is based on public key logic and encryption to secure shared patient data [18,19,51]. Therefore, the solution remains patient-centric as the patient holds the necessary private key token to allow the provider to tap into the on-chain metadata and access her or his off-chain health data kept on the backend cloud storage. While the broadly known blockchain solutions of cryptocurrencies and non-fungible-tokens (NFTs) use a public chain code, allowing every individual to access and participate, the hereby presented technological concept is built upon a permissioned blockchain-based system [49,52]. Therefore, only selected network providers (i.e., the senological oncological care centers or doctors) have the ability for data sharing and integration, but only if the patient uses her private key to enable that specific provider to access the data and add to them. Each service supplier provides a blockchain node integrated with its hospital information system to form the blockchain network. The patient her- or himself is presented by a web- or app-based interface used to facilitate data sharing transactions between doctor and patient. The technological solution is depicted by Figure 3 and presented by further explanation in the following.

#### 3.3.4. Cloud Storage

The technological solution is based on a hybrid data storage structure with metadata stored on-chain while sensitive patient data are saved and managed in a GDPR-compliant cloud storage as a backend solution [49]. This provides high scalability, high availability, and low latency.

#### 3.3.5. Web Application

Patients and providers are presented with an easy-to-use web- and app-based frontend solution. This enables uncomplicated communication between the stakeholders (i.e., caregiver and patient) and the chain code server. Fundamentally, it is not crucial for patients and providers to understand the underlying technological solution, but rather its core principles and leveraged usability for daily care.

#### 3.3.6. Blockchain Nodes

The backend cloud-based data storage relates to the frontend web- or app-based solution via blockchain logic. Each senological care center has the authorization to act as a system node within the code logic and hash metadata to the ledger.

#### 3.3.7. Cryptographic Operations

The hybrid cryptosystem will allow patient data encryption before uploading to the chain code server, which can then be accessed by caregivers based on permissions and be granted by the patient (stored on the ledger) to access specific patient data. As a result, the doctors can only access the data based on the allowance by the patient. This leverages patient data privacy and confidentiality. The DLT or blockchain properties offer the possibility to protect the sensitive personal data that flow in the system [49].

To put it in a nutshell, a patient accesses the web platform via an app-based application that he or she is already familiar with from daily tablet or smartphone use. The patient can then share their personal health data with a specific certified service provider. Here, the patient chooses the length of time and content scope of the data sharing. At the same time, the health data undergo encryption before being uploaded to the ledger. This ensures that only unreadable metadata are on the ledger, while the sensitive data remain on the cloud storage. A care provider can now log into the hybrid system via the application as well. Based on the permissions, which were previously defined by the patient, health data can be viewed by a caregiver. A consideration of the right-to-be-forgotten can be realized at this point by the deletion of data from the cloud storage. This is possible as the metadata on the chain remain “contentless” as they reflect exclusively the transaction history in the ledger. Thus, touchpoints between stakeholders can be reproduced in principle, but not their content. In addition, each patient retains the ability to revoke authorizations to care providers at any time.

## 4. Discussion

### 4.1. Challenges and Pain Points of Senological Oncological Care

Not only oncological care, but whole healthcare systems are and will be facing various challenges in order to provide sufficient and efficient care in the near future. Digital solutions that aim at lifting obligations from providers while enhancing quality and efficiency of care for the patient need to tackle challenges and find solutions for pain points for medical service provision on multiple levels [19,21,35].

#### 4.1.1. Interoperability and Accessibility

Patient data including the personal health history, diagnostic results, or treatment protocols provide the necessary information base for physicians to make informed medical decisions in senological oncological and gyne-oncological care. Timely sharing of a patient’s health data across providers facilitates efficient medical service provision. Status quo oftentimes holds onto a manual process to transfer their individual health information from one care provider to another. Therefore, a paper-based consent form specifying the extent and type of data that will be shared needs to be signed in advance [19,23]. A lack of standard systems architecture fails to establish security and data sovereignty of patients once the data are shared. This also makes health data sharing very tedious and time-consuming, with doctors spending more time on gathering sensitive information over phone, fax, or mail then on actual treatment. These cumbersome processes tend to lead to major delays in patient care. Digital solutions need to make use of network effects to overcome siloed data storage and leverage interoperability and accessibility [20].

#### 4.1.2. Privacy, Security, and Data Integrity

Moreover, the data are highly private and sensitive; therefore, the patient or their caregiver, where required, should have the right to be able to set rules and limit access to the data. The current system keeps the patient out of the loop and the data stored in siloed hospital information systems [18]. Therefore, the data management of current breast cancer care fails to establish patient centrism and data sovereignty.

#### 4.1.3. Process Complexity

The outline of the breast cancer care workflow provides a holistic overview of the high process complexity of diagnostics, treatment, and follow-up care. Imaginably, the patient faces a high information asymmetry towards the doctor regarding educated decision making on the personal treatment. On the other side, the doctor is in need of decision support systems in order to select the best treatment option as oncological care research, especially in the case of breast cancer, is developing at a rapid pace. Thus, digital solutions need to support the doctor in decision support to provide the optimum quality of care while the patient needs assistance along their patient journey to lower information asymmetry and leverage the patient’s treatment compliance [19,23,28].

#### 4.1.4. Documentation Obligations

Documentation plays a significant role in healthcare service provision. It provides a comprehensible base for doctors to be able to reproduce the colleagues’ work while securing forensic safety of treatment. Unfortunately, this legal safety has become more and more important as trust in care has decreased over the past years and, therefore, the importance of sufficient documentation has risen to a nearly unbearable level for doctors [13]. As a result, doctors spend a significant share of their worktime on this issue and less and less time on direct patient care. Digital solutions need to lift these obligations from doctors via automated documentation to free up worktime and refocus work efforts on direct medical care.

#### 4.1.5. Shifting Demographics and Work Environment

These developments are intensified by shifting demographics and an impairing work environment. In 2020, for example, only 19.1% of all doctors working in Germany were younger than 35 years of age, in comparison with 24.8% in 1995 [53]. The medium age of the doctoral work force is on a constant rise, leaving 59.6% of gynecological and obstetric specialists with an age of 50 years or higher [53]. Furthermore, 83.4% of all doctors completing their residency specialization in gynecology and obstetrics in Germany in 2020 were female [53]. Thus, gynecology and obstetrics is leading by example and will arrive in a female-dominated state that logically and thankfully calls for a change in work environment, enabling flexible working conditions, maternity leaves, and a compatibility of family and work. Therefore, digital solutions need to streamline these developments to prevent a supply bottleneck of doctoral service provision.

#### 4.1.6. Rising Economic Pressure and Increasing Case Turnover

Ultimately, economic pressure is on a constant rise, as total national healthcare spending is growing rapidly, with 410.8 bn€ in 2019 in comparison with 281.6 bn€ in 2009 [54]. Therefore, health expenses made up 11.9% of national GDP (gross domestic product) in 2019 [54]. On the other hand, the average length of stay has decreased by 27.3% and the overall case number has increased by 12% in a 20-year observation period from 1999 to 2019 [54]. Thus, digital solutions need to drive partial atomization in order to handle the combination of increasing case turnover and diminished workforce while leveraging process efficiency to slow down the swift cost increase [39].

### 4.2. Use Case Potential of Breast Cancer Care

Invasive mammary carcinoma presents as the leading type of female cancer disease in Germany. The yearly national incidence amounts to over 70,000 primary diagnoses and can predominantly be seen in females, with a relative coverage of 99% of overall cases [55]. About 45% of all primary lesions are discovered within the screening age group of 50–69 years, while 18% and 37% are represented by the age groups of <50 and ≥70, respectively [55]. Therefore, the hereby presented descriptive data of the regional breast cancer care network of Marburg University Hospital present as comparable to national statistics with regard to age and gender distribution. Fortunately, the 5-year survival rate of C50 invasive breast cancer amounts to 88%, in comparison with the second most frequent cancer type of colorectal cancer (C18-21) with 66%. Furthermore, C18-21 presents an average age of disease onset of 72.9, while invasive breast cancer displays a comparably young group of patients of 64 [55].

Besides the pure age-related reasoning, invasive breast cancer presents a comparably “healthy” patient collective. In the previously presented descriptive comparison to ovarian cancer, breast cancer presents a low case complexity level of 0.5, an average of 6.2 procedures performed on each patient, and 2.7 secondary diagnoses coded for each breast cancer patient. Meanwhile, ovarian cancer displays a more morbid patient collective, with an average PCCL of 2.4, 9.2 secondary diagnoses, and 9.0 procedures performed on each ovarian cancer patient.

All of the necessary data for decision making along the care process (i.e., mammography, mamma ultrasound, histopathologic result of mamma biopsy, medical history, transvaginal ultrasound, or blood count) would be stored on the blockchain or distributed ledger network, while the patient is guided along the patient journey through AI-enabled monitoring. In this case, it is not important which provider hashes the blocked data as they are securely stored within the network, i.e., a patient running her diagnostics in a senological oncological care center in Munich keeps the possibility to present her data for the treatment decision at the care center in Marburg. The descriptive data have shown the high level of regional centrism of breast cancer care. In 2021, 96% of all admitted breast cancer cases of Marburg University Hospitals presented a zip code starting with the digits of 34, 35, or 36. Thus, the majority of patients show direct geographical proximity to Marburg, calling for a smooth switch between the various in- and out-patient providers within the network. Furthermore, once a patient leaves the regional network, almost all data will be kept in the siloed infrastructure and, for external caregivers, the data gathering process will present as even more cumbersome as regional specifics are unknown in advance. For example, a doctor at a hospital in Munich has no knowledge on how and whom to call in the regional Marburg network to gather necessary treatment information and, as a result, this process will consume extra effort and time. Even if a patient faces a relapse after more than 10 years and the patient may have relocated geographically, i.e., from Marburg to Munich, she can share all of the information of her initial cancer treatment via private key facilitated allowance to the new senological oncological care center to access the information. As invasive breast cancer presents a comparably young patient collective with an average age of 61.4 within the previously presented descriptive analysis of the Marburg University Hospital treatment data, the foreseen long-term tertiary prevention of a minimum of 10 could cause challenges once a patient relocates and experiences a relapse. In addition, the estimated circumference of the C50 cases being 28.6% shorter than for C56 cases and the estimated total catchment area being 40% smaller for breast cancer care in descriptive statistics for Marburg University Hospital underline the high level of regional centrism of breast cancer care.

Therefore, we identify breast cancer treatment as a suitable area of implementation for modern technologies as a large rate of digital immigrants and digital natives among the clientele can be expected as well as high survival rate conditions for long-term secondary treatment and tertiary prevention monitoring that needs to be independent from geographical boundaries [56,57]. Furthermore, the descriptive analysis describes a “healthier” patient collective in comparison with ovarian cancer; therefore, we expect far more active personal treatment planning and monitoring participation by breast cancer patients. This lays the basis for the use case potential of the digital workflow concept.

### 4.3. Dualistic Technological Solution Approach

Ultimately, the previously presented digital workflow concept tackles the identified challenges and pain points of the current breast cancer patient journey by making use of a dualistic technological solution approach.

#### 4.3.1. Artificial Intelligence

Artificial intelligence uses algorithmic standardized solution findings to accompany the patient along the workflow. It partially replaces the doctor at times where she or he is not able to help owing to increased work intensity [48]. It is the key to surpass the process complexity of oncological care, leveraging ease of use and granting successful treatment compliance [6].

On the other hand, it is a valuable tool for the doctor. Through automated documentationm it frees up the provider’s time; via decision support, it enhances quality of care; and, as a result, worktime is freed up towards direct patient care and the empathetic communication, which a digital solution cannot guarantee to a full extent [37]. Thus, it has the capacity to tackle the developments of shifting demographics and improve the work environment of healthcare workers [8]. As it paves the way towards partial automatization, it empowers the doctor to redirect her or his efforts to the core of medicine, that is, empathetic and high-quality care for the patient [29].

#### 4.3.2. Blockchain or Distributed Ledger Technology

The underlying distributed ledger technology originated from blockchain and cryptocurrencies like Bitcoin and finds its application in healthcare as it enables decentralized database solutions to tackle current challenges of healthcare provision while complying with the European Union’s General Data Protection Regulation (GDPR) and ensuring the user’s data security and privacy [11,13,15,19,21]. This enables efficient, effective, and timely service provision by the providers, as the permissioned private blockchain solution tears down the core challenge of lacking interoperability and service interface problems within the provider network [20].

A dualistic technological approach will speed up efficiency to face the rising economic pressure as it helps to cover the increased turnover in patient care. As a result, this drives the potential of the presented concept to add economic value on a microeconomic level for service providers within the senological oncological care network and on a macroeconomic level for the entire healthcare system, while providing a better quality of care for the patient.

### 4.4. Benefits

The core benefits for patients and providers of the hereby presented workflow are presented by four key pillars.

#### 4.4.1. Ease of Use

Historically, a barrier to entry for decentral application by non-technical and novice users has been the lacking usability of the solution. The combination with artificial intelligence as a dualistic model overcomes these adaption hurdles. Ease of use is created by the AI-based user experience, and the benefits created by developing a digital decision support assistant, treatment planner, and companion that helps out doctors at times fail to do so. Vice versa, providers profit from a partially automated workflow and are able to refocus on the core of their profession. In the end, the perceived usability of the digitized breast cancer care for patients and providers could mostly be provided by the underlying AI-functionality, but in order to realize its full medical and economic potential, pairing with a data sharing solution is key.

#### 4.4.2. Interoperability

The hybrid model of a decentralized distributed ledger technology, based on a permissioned private blockchain, in combination with a cloud-based backend data storage helps to overcome the current siloed data structure and provides the efficiency of the model needed to realize its full medical and economic potential as it provides the ability of the different computer systems to connect and exchange information with each other without restriction. As such, the information can smoothly move across from provider to provider with minimal friction while complying with security and privacy standards.

#### 4.4.3. Network Effects

A network effect is a phenomenon whereby large numbers of users or participants increase a service’s value. Thus, the true value of the proposed model will be realized once the whole German breast cancer network of about 280 DKG-certified (Deutsche Krebsgesellschaft, German Cancer Association) senological care centers participate. Ultimately, benefits will be created for every participant within the network as overall efficiency will be leveraged. The concept redirects the efforts to the whole network and does not favor a specific stakeholder group.

#### 4.4.4. Shared Governance

The current system leaves patients nearly entirely out of the loop regarding educated decision making and data sovereignty. The key cryptography of distributed ledger technology places the patient in a powerful position. Personal data sovereignty is redirected into the patients’ hands. On the other hand, AI-based decision support and information lowers the patient–doctor information asymmetry and enables educated shared decision making for treatment.

### 4.5. Limitations and Future Research

The study design relies on the retrospective analysis of single-center data. Thus, transferability and generalization of the results are limited. Data refer to rather rural environments that compare to Marburg-Biedenkopf county and its surroundings. It remains unclear whether urban centers will display comparable results. This particular type of data generation restricts the study layout to the analysis of inpatient cases. Given that data generation is based on ICD codification, effects of erroneous coding, incorrect diagnoses, or possible effects due to modest changes in ICD categorization during the observational period cannot be dismissed. For the aforementioned reasons, an expansion of the analysis to a more urban setting, an outpatient environment, and a multicenter data comparison would be beneficial.

The literature used for the development of the digital concept is limited to the extent that is focuses on a holistic industry assessment. It lacks a clear depiction on technology readiness and diffusion. Therefore, further research remains necessary to review the state-of-the-art standard of artificial intelligence and distributed ledger technology in the fields of oncology or, more specifically, gyne-oncology by identifying suitable primary literature. The current literature fails to answer the technology readiness question of whether viable solutions in the stage of concepts, designs, or prototype are existent.

As the proposed model is built upon the connection of existing solutions via application programming interface, the technological realization is maximized regarding cost-efficiency and feasibility. Nonetheless, it remains questionable whether acceptance and adaption can be successfully realized within the proposed senological care network with a model based on modern technologies like artificial intelligence and distributed ledger technology. Thus, pre-launch empiric questioning remains necessary to assess patients’ and providers’ satisfaction with the current non-digital processes and to measure their perceived benefits and the potential of model realization. The question regarding adaption or rejection remains open. Further research needs to utilize technology acceptance modeling in order to credibly respond to this key question before investing into and implementing a pilot project [58].

## 5. Conclusions

We identify invasive breast carcinoma, the leading type of female cancer disease, as a suitable digitization use case for artificial intelligence as well as blockchain and distributed ledger technology application. The combination of its comparably young average age of disease onset, low patient complexity and number of secondary diagnoses, as well as the high five-year survival rate offer a great potential for the digitization of a long-term patient care plan of at least ten years. The patient collective suggests a high number of digital immigrants and natives prone to using digital app-based solutions based on an adequate capability of making use of digitally supported disease accompaniment.

Furthermore, the frequency and geographical distribution visualization promote a comparably high degree of regional centralization of breast cancer care. Therefore, interconnectedness within the regional network as well as cross-regional information exchange can be leveraged by decentralization of the current siloed data infrastructure.

The prevailing challenges and trends of lacking interoperability and accessibility, low privacy and data integrity standards, high process complexity in combination with rising documentation obligations, a shifting demographic and work environment, as well as rising economic pressure due to increasing case turnover are likely to intensify in the near future. The proposed dualistic digital transformation concept of artificial intelligence and distributed ledger technology, based on a hybrid data management approach of a permissioned private blockchain network and privacy-compliant cloud storage, indicates great potential towards addressing these pain points. The digitization of the breast cancer care workflow creates benefits for the patients, providers, and entire health care system by leveraging the ease of use and interoperability while utilizing multiple network effects and establishing a shared governance structure that equips the patient with data sovereignty and moves her into the center of caregiving.

Further research needs to evaluate the transferability of findings to urban environments and multicentric evaluation to provide a clear depiction on technology readiness and diffusion. The question regarding adaption or rejection remains open and, therefore, empiric testing and technology acceptance modeling are necessary to credibly respond to this key question before investing into and implementing a pilot project.

## Figures and Tables

**Table 1:** Results of the C50 and C56 patient data analysis.

	Absolute Figures							
**C50**	**2017**	**2018**	**2019**	**2020**	**2021**	**5-year average**	**Average Age (2017–2021)**	
**Patient** **number**	407	424	461	429	468	437.8	**Male**	71.3
**LoS**	5.3	5.3	4.8	5.1	4.7	5.0	**Female**	61.4
**PCCL**	0.5	0.5	0.5	0.5	0.4	0.5		
**SD**	2.0	2.3	2.0	2.8	4.7	2.7		
**PROC**	7.0	6.7	6.0	5.9	5.2	6.2		
**C56**	**2017**	**2018**	**2019**	**2020**	**2021**	**5-year average**	**Average Age (2017–2021)**	
**Patient** **number**	72	81	85	73	92	80.6	**Female**	59.9
**LoS**	12.2	13.0	13.6	13.4	12.8	13.0		
**PCCL**	2.3	2.6	2.5	2.1	2.5	2.4		
**SD**	6.3	7.8	8.0	9.4	14.8	9.2		
**PROC**	8.4	8.8	9.3	8.8	9.9	9.0		

LoS = length of stay in days, PCCL = patient clinical complexity index, SD = average number of secondary diagnoses, PROC = average number of procedures performed on each patient.

**Table 2:** C50 frequency distribution by two-digit zip codes.

Two-Digit Zip Code	2017	2018	2019	2020	2021	Sum	2017 Relative Distribution	2021 Relative Distribution
**34**	45	29	33	37	40	184	11%	9%
**35**	307	341	353	333	370	1.704	75%	79%
**36**	35	28	50	37	38	188	9%	8%
**57**	4	7	9	7	8	35	1%	2%
**Others**	16	19	16	15	12	78	4%	3%
**Sum**	**407**	**424**	**461**	**429**	**468**	**2.189**	**100%**	**100%**
**Counties** **starting with**	**34**		**35**		**36**		**57**	
	− Kassel − Schwalm-Eder-Kreis − Göttingen − Höxter − Hochsauerlandkreis − Waldeck-Frankenberg		− Marburg-Biedenkopf − Waldeck-Frankenberg − Gießen − Vogelsbergkreis − Lahn-Dill-Kreis − Wetteraukreis − Limburg-Weilburg		− Fulda − Vogelsbergkreis − Hersfeld-Rotenburg − Werra-Meißner-Kreis − Schwalm-Eder-Kreis − Main-Kinzig-Kreis − Wartburgkreis − Schmalkalden-Meiningen		− Siegen-Wittgenstein − Olpe − Hochsauerlandkreis − Westerwaldkreis − Neuwied	

**Table 3:** Results of the Boolean combination search.

{(“Distributed Ledger” OR Blockchain) AND (Medicine OR Oncology)}			
Index	Title	Year of Publication	Authorship Institution
**1**	A prescription for blockchain and healthcare: Reinvent or be reinvented [11]	2018	PwC
**2**	A prescription for blockchain in healthcare [12]	2018	BCG
**3**	Blockchain—Use in the German healthcare system (Blockchain Einsatz im deutschen Gesundheitswesen) [13]	2017	Deloitte
**4**	Blockchain in health [14]	2016	Ernst and Young
**5**	Blockchain in healthcare [15]	2020	Frankfurt School of Finance
**6**	Blockchain opportunities for patient data donation and clinical research [16]	2018	Deloitte, Pfizer
**7**	Blockchain to blockchains in life sciences and health care [17]	2018	Deloitte
**8**	Blockchain: the democratization of healthcare (Blockchain: Die Demokratisierung des Gesundheitswesens?) [18]	2017	WIG
**9**	Blockchain: The chain of trust and its potential to transform healthcare—Our point of view [19]	2016	IBM
**10**	Demystifying blockchain for life sciences: blockchain could be a key to interoperability and privacy [20]	2018	KPMG
**11**	Healthcare rallies for blockchains [21]	2016	IBM
**12**	In Blockchain we trust: transforming the life sciences supply chain [22]	2018	Accenture
**13**	Opportunities and challenges of blockchain technologies in health care [23]	2020	OECD
**14**	Prescribing a paperless society: how blockchain can deliver electronic prescriptions [24]	2017	PwC
**15**	The internet of things and blockchain: unique opportunities for healthcare [25]	2018	Oracle
**{(“Artificial Intelligence” OR “Machine Learning”) AND (Medicine OR Oncology)}**			
**Index**	**Title**	**Year of Publication**	**Authorship Institution**
**1**	A smarter way for healthcare companies to go digital [26]	2020	Bain & Company
**2**	Artificial intelligence in global health [27]	2019	USAID
**3**	Artificial intelligence in healthcare: past, present and future [28]	2017	SVN
**4**	Artificial intelligence: healthcare‘s new nervous system [6]	2017	Accenture
**5**	Chasing value as AI transforms health care [29]	2019	BCG
**6**	Contribution to the discussion on the European Commission’s data strategy and AI white paper [30]	2020	eit Health
**7**	Digital and physical innovations stimulate the healthcare sector [31]	2021	Roland Berger
**8**	Digital transformation—Shaping the future of European healthcare [32]	2020	Deloitte
**9**	Digital transformation—Where does the German healthcare system stand? (Digitale Transformation—Wo steht das deutsche Gesundheitswesen?) [33]	2020	Deloitte
**10**	Future of health: an industry goes digital—Faster than expected [34]	2019	Roland Berger
**11**	Artificial intelligence—Revolution for the healthcare industry (Künstliche Intelligenz—Revolution für die Gesundheitsbranche) [35]	2018	BVDW
**12**	Mind the (AI) gap—Leadership makes the difference [36]	2018	BCG
**13**	With artificial intelligence to clinical and operational success (Mit künstlicher Intelligenz zum klinischen und betrieblichen Erfolg [37]	2018	Philips
**14**	AI and Health Project Group—Summary of preliminary results (Projektgruppe, KI und Gesundheit—Zusammenfassung der vorläufigen Ergebnisse) [38]	2019	Deutscher Bundestag
**15**	Sherlock in health—How artificial intelligence may improve quality and efficiency, whilst reducing healthcare costs in Europe [39]	2017	PwC
**16**	The potential for artificial intelligence in healthcare [8]	2019	Future Healthcare
**17**	Transforming healthcare with AI—The impact on the workforce and organizations [40]	2020	McKinsey & Company
**18**	White paper on artificial intelligence—A European approach to excellence and trust [41]	2020	European Commission
**19**	Whitepaper for the ITU/WHO focus group on artificial intelligence for health [42]	2020	ITU/WHO

## Data Availability

The datasets generated and analyzed during the current study are available from the corresponding author upon reasonable request.
